# Magnitude of and Characteristics Associated With the Treatment of Calcium Channel Blocker–Induced Lower-Extremity Edema With Loop Diuretics

**DOI:** 10.1001/jamanetworkopen.2019.18425

**Published:** 2019-12-27

**Authors:** Scott Martin Vouri, Xinyi Jiang, Todd M. Manini, Laurence M. Solberg, Carl Pepine, Daniel C. Malone, Almut G. Winterstein

**Affiliations:** 1 Department of Pharmaceutical Outcomes and Policy, University of Florida College of Pharmacy, Gainesville; 2 Center for Drug Evaluation and Safety, University of Florida, Gainesville; 3 Department of Aging and Geriatric Research, University of Florida College of Medicine, Gainesville; 4 Geriatric Research, Education, and Clinical Center, Malcom Randall North Florida/South Georgia VA Medical Center, Gainesville, Florida; 5 Division of Cardiovascular Medicine, Department of Medicine, University of Florida College of Medicine, Gainesville; 6 Department of Pharmacy Practice and Science, University of Arizona College of Pharmacy, Tucson; 7 Department of Epidemiology, University of Florida College of Medicine, Gainesville; 8 College of Public Health and Health Professions, University of Florida, Gainesville

## Abstract

**Importance:**

Calcium channel blockers, specifically dihydropyridine calcium channel blockers (DH CCBs, eg, amlodipine), may cause lower-extremity edema. Anecdotal reports suggest this may result in a prescribing cascade, where DH CCB–induced edema is treated with loop diuretics.

**Objective:**

To assess the magnitude and characteristics of the DH CCB prescribing cascade.

**Design, Setting, and Participants:**

This cohort study used a prescription sequence symmetry analysis to assess loop diuretic initiation before and after the initiation of DH CCBs among patients aged 20 years or older without heart failure. Data from a private insurance claims database from 2005 to 2017 was analyzed. Use of loop diuretics associated with initiation of angiotensin-converting enzyme inhibitors, angiotensin receptor blockers, and other commonly used medications was used as negative controls. Data were analyzed from March 2019 through October 2019.

**Exposures:**

Initiation of DH CCB or negative control medications.

**Main Outcomes and Measures:**

The temporality of loop diuretic initiation relative to DH CCB or negative control initiation. Secular trend-adjusted sequence ratios (aSRs) with 95% CIs were calculated using data from 360 days before and after initiation of DH CCBs.

**Results:**

Among 1 206 093 DH CCB initiators, 55 818 patients (4.6%) (33 100 [59.3%] aged <65 years; 32 916 [59.0%] women) had a new loop diuretic prescription 360 days before or after DH CCB initiation, resulting in an aSR of 1.87 (95% CI, 1.84-1.90). An estimated 1.44% of DH CCB initiators experienced the prescribing cascade. The aSR was disproportionately higher among DH CCB initiators who were prescribed high doses (aSR, 2.20; 95% CI, 2.13-2.27), initiated amlodipine (aSR, 1.89; 95% CI, 1.86-1.93), were men (aSR, 1.96; 95% CI, 1.91-2.01), and used fewer antihypertensive classes (aSR, 2.55; 95% CI, 2.47-2.64). The evaluation of ACE inhibitors or ARBs as negative controls suggested hypertension progression may have tempered the incidence of the prescribing cascade (aSR for ACE inhibitors and ARBs, 1.27; 95% CI, 1.24-1.29).

**Conclusions and Relevance:**

This study found an excessive use of loop diuretics following initiation of DH CCBs that cannot be completely explained by secular trends or hypertension progression. The prescribing cascade was more pronounced among those initially prescribed a high dose of DH CCBs.

## Key Points

**Question:**

What is the risk of experiencing the prescribing cascade of treating dihydropyridine calcium channel blocker–induced lower-extremity edema with a loop diuretic?

**Findings:**

In this cohort study of 1.2 million patients who initiated a dihydropyridine calcium channel blocker, excessive use of loop diuretics was found, which cannot be fully explained by secular trends or hypertension progression. This was particularly pronounced among patients who received a high dose of dihydropyridine calcium channel blockers.

**Meaning:**

In this study, many patients received loop diuretics instead of a dose reduction or discontinuation of dihydropyridine calcium channel blockers, constituting a prescribing cascade. Future studies are needed to test strategies to mitigate or prevent prescribing cascades.

## Teaser

This cohort study used prescription sequence symmetry analysis to assess the magnitude and characteristics of the prescribing cascade of treating dihydropyridine calcium channel blocker–induced lower-extremity edema with loop diuretics.

## Introduction

Hypertension is the most common chronic condition in the United States, occurring in nearly half of all adults.^1^ Dihydropyridine calcium channel blockers (DH CCBs) are prescribed to approximately 1 in 5 adults with hypertension in the United States^2^ and are considered a first-line option given their cardiovascular benefits.^3,4,5,6^ Dihydropyridine calcium channel blockers are also generally considered safe because they do not require routine electrolyte or kidney function monitoring, nor do they cause diuresis.^7,8,9,10^ A disadvantage to their use is the risk of lower-extremity edema, a dose-dependent and duration-dependent adverse event with an estimated incidence of 12%.^10,11,12^ The preferred treatment for DH CCB–induced edema includes DH CCB dose reduction or discontinuation, which typically reduces or completely resolves the edema.^13^

Although not recommended, a loop diuretic can be used to symptomatically treat DH CCB–induced edema.^14^ Use of a loop diuretic for this purpose constitutes a prescribing cascade, in which a drug-induced adverse event prompts additional medication treatment rather than discontinuing or reducing the original prescription.^15^ This prescribing cascade can be classified as problematic prescribing^16^ because it not only results in the use of additional medications (eg, potassium supplements) and thus exacerbates polypharmacy^17^ but can also lead to preventable adverse events (eg, acute kidney injury, severe dehydration, increased urinary frequency or incontinence, hypotension, fall-related injuries, and electrolyte abnormalities that may result in arrhythmias).^18,19,20,21,22,23,24,25,26,27^

With a dearth of evidence available regarding the prescribing cascade, including a case report^28^ and a study^29^ that used cross-sectional data, as well as the risk of poor health outcomes and increased health care utilization associated with the prescribing cascade,^30^ there is a need for more extensive research. Accordingly, our primary objective was to evaluate the risk of this prescribing cascade using prescription sequence symmetry analysis (PSSA). Our secondary objective was to explore the incidence of the prescribing cascade among subpopulations.

## Methods

### Design

We used a PSSA to evaluate the temporality of the initial loop diuretic prescription relative to the initial DH CCB prescription. This pharmacovigilance approach, a case-only design first published by Hallas^31^ in 1996 and subsequently validated in 165 medication pairs with a sensitivity of 61% and a specificity of 93%,^32^ has since been used to assess the presence of multiple prescribing cascades in several studies.^33^ Among patients initially prescribed the medication suspected of causing a drug-induced adverse event (ie, index drug; in this case, a DH CCB) and the medication potentially used to treat the adverse event (ie, marker drug; in this case, a loop diuretic), PSSA assesses the timing of the initial marker drug relative to initial index drug.^34^ A similar number of patients would be prescribed the marker drug before and after the index drug if the index drug was not associated with the use of the marker drug, resulting in a symmetrical pattern when displayed graphically. However, in the case of a prescribing cascade, where the marker drug is used to treat the adverse event of the index drug, a higher proportion of initiations of the marker drug would occur after the initiation of the index drug compared with before. Because PSSA is a type of within-patient analysis, time-invariant confounders are inherently adjusted^32,35^; however, stratified analyses can be used to assess the prescribing cascade among subpopulations.

### Data Source

We used the MarketScan Commercial and Medicare Supplemental Claims databases (IBM Corp) data from January 2005 to December 2017. These nationwide administrative claims databases contain deidentified person-level information on health care utilization and enrollment records across all settings, including outpatient visits, hospital stays, and pharmacy claims. The study population included employees, dependents, and retirees with employer-sponsored or Medicare Supplemental insurance. The study was exempted from review by the University of Florida institutional review board because of its use of deidentified data. We used the Strengthening the Reporting of Observational Studies in Epidemiology (STROBE) reporting guideline to ensure appropriate reporting.^36^

### Population

Calcium channel blocker initiators were identified among patients aged 20 years or older with at least 720 days of continuous enrollment before and 360 days after an initial DH CCB claim.^37^ Patients with a heart failure diagnosis on inpatient or outpatient encounters within 720 days before and 360 days after the initial DH CCB claim were excluded because the use of a loop diuretic among these patients may have been for heart failure–related fluid overload^38,39^ (eTable 1 in the Supplement). Next, we identified the initial loop diuretic claim within 360 days before or after the initial DH CCB claim because use of a loop diuretic beyond this window is likely not attributable to DH CCB–induced edema.^10^ Using this exposure window also limited the effect of within-patient time-varying bias (ie, loop diuretics are generally used later in life or later in an antihypertensive regimen, suggesting hypertension progression) that may occur if larger exposure windows were used.^34,40^ Similar to previous studies, patients were excluded if initial claims of both index and marker drugs occurred on the same date.^41,42^

### Population Assessment

We performed stratified analyses among subpopulations based on age (ie, <65 and ≥65 years) because older adults may be more likely to experience a prescribing cascade because of polypharmacy, increased complexity of clinical management of multiple chronic conditions, and increased susceptibility to adverse events^29^; sex because women are more likely to report edema^13,22,43^; initial DH CCB type (ie, amlodipine, isradipine, nifedipine, or felodipine) because amlodipine is associated with an increased risk of edema^10^; initial DH CCB dose (ie, low, standard, or high) (eTable 2 in the Supplement) because risk of DH CCB–induced edema increases with higher doses^11,12,44,45,46,47^; and number of unique antihypertensive medication classes used within 1 year before DH CCB initiation because a loop diuretic may be used if multiple antihypertensive medication classes have failed to control hypertension.^48^

### Statistical Analysis

We calculated the crude sequence ratio (cSR) by dividing the number of patients with the initial marker drug claim after the initial index drug claim by the number of patients with the initial marker drug claim before the initial index drug claim. The cSR was represented graphically by assessing the initial loop diuretic claim within 360 days before and after the initial DH CCB claim in 30-day increments, similar to previous PSSA studies.^34,40,49^

To adjust for secular trends in medication use (ie, increasing or decreasing use of loop diuretics or DH CCBs during the study period), we calculated the null-effect ratio,^31^ following the approach by Takeuchi et al.^50^ An adjusted sequence ratio (aSR) with 95% CIs was then calculated by dividing the cSR by the null-effect ratio.^51^ We considered nonoverlapping CIs to be indicative of a statistically significant difference.

We estimated the cumulative annual incidence of DH CCB initiators with the prescribing cacade.^49^ In operationalizing the definition of the prescribing cascade, we assumed the proportion of patients with loop diuretic initiation after DH CCB initiation in excess of the proportion of patients with loop diuretic initiation before DH CCB initiation to be prescribing cascade–associated.^33^ Accordingly, to determine the incidence of the prescribing cascade among all DH CCB initiators, we calculated the difference between the number of patients with the initial loop diuretic claim after the initial DH CCB claim and the number of patients with the initial loop diuretic claim before the initial DH CCB claim and then divided the result by the total number of DH CCB initiators. We also estimated the incidence of the prescribing cascade among DH CCB initiators in 3 periods (ie, 2007-2010, 2011-2013, and 2014-2016) and among the previously described subpopulations using stratum-specific incidence estimates.

To evaluate within-patient, time-varying bias (eg, loop diuretics are generally used later in life or later in an antihypertensive regimen), we performed PSSA for the initiation of loop diuretics among patients without a heart failure diagnosis who were prescribed either angiotensin-converting enzyme (ACE) inhibitors or angiotensin receptor blockers (ARBs), first-line antihypertensive classes. Because DH CCBs can be prescribed before or after the prescription of an ACE inhibitor or ARB, we excluded patients with DH CCB use within 360 days before or after initiation of the negative control index drugs. We also evaluated initiators of other commonly used medications as index drugs, including levothyroxine, tiotropium, and nonbenzodiazepine hypnotics (eg, eszopiclone, zaleplon, zolpidem), among patients without a heart failure diagnosis and without DH CCB use 360 days before or after the initiation of negative control index drugs. These alternative index drugs served as negative controls because they are not associated with edema and would have similar health care follow-up after initiation to ensure similar opportunities for edema diagnosis and loop diuretic prescribing.^52^ Any increase in loop diuretic prescribing following the initiation of negative controls (especially an ACE inhibitor or ARB) could potentially be explained as hypertension progression, with loop diuretics being used to control blood pressure. Secondary analyses were conducted among negative controls stratified by age (ie, <65 years vs ≥65 years) and number of other antihypertensive medications (ie, 0-1, 2-3, or ≥4) because the timing of loop diuretic initiation may differ within these subpopulations. In a post hoc analysis, we reanalyzed the initiation of loop diuretics restricted to 180 days before and after DH CCB, ACE inhibitor, or ARB initiation to further mitigate within-patient, time-varying biases (eg, hypertension progression).

All analyses were conducted using SAS statistical software version 9.4 (SAS Institute). The hypothesis was 2-sided with an α < .05 considered statistically significant. Data were analyzed from March 2019 through October 2019.

## Results

**Figure 1.**
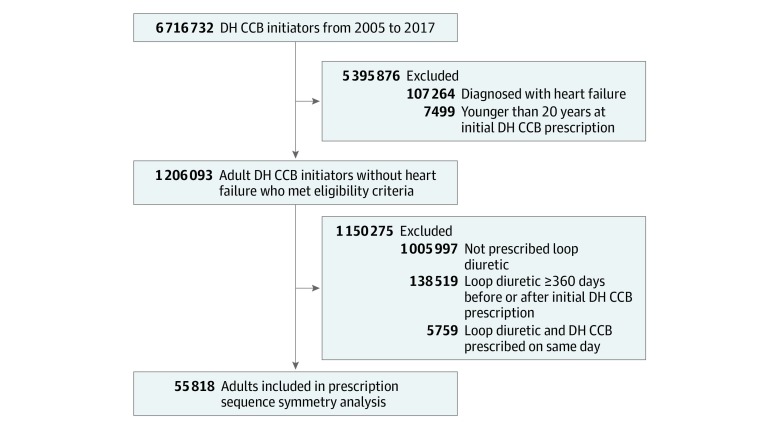
Flow Diagram for Identifying the Prescribing Cascade DH CCB indicates dihydropyridine calcium channel blocker.

We identified 6 716 732 unique DH CCB initiators. After applying health plan enrollment criteria and restrictions, 1 206 093 DH CCB initiators remained (Figure 1). After additional exclusions, we included 55 818 patients (33 100 [59.3%] aged <65 years; 32 916 [59.0%] women) in the PSSA. Because the PSSA required the use of DH CCBs and loop diuretics, the included population differed from the overall population of DH CCB initiators. Specifically, a higher proportion of patients included in the PSSA than those excluded had their index prescription between 2007 and 2010 (25 804 [46.2%] vs 470 988 [40.9%]), were aged 65 years or older (22 718 [40.7%] vs 289 942 [25.2%]), were women (32 916 [59.0%] vs 582 294 [50.6%]), were initially prescribed high-dose DH CCBs (17 310 [31.0%] vs 248 519 [21.6%]), and received at least 4 other antihypertensive medications (8613 [15.4%] vs 77 765 [6.8%]) (eTable 3 in the Supplement).

Amlodipine was the most commonly initiated DH CCB (49 930 initiators [89.5%]) with 6684 [12.0%], 31 742 [57.0%], and 17 310 [31.0%] individuals prescribed a low, standard, and high dose, respectively. The mean (SD) number of antihypertensive classes (excluding loop diuretics) in the 2 years before the initial DH CCB was 2.17 (1.33).

**Figure 2.**
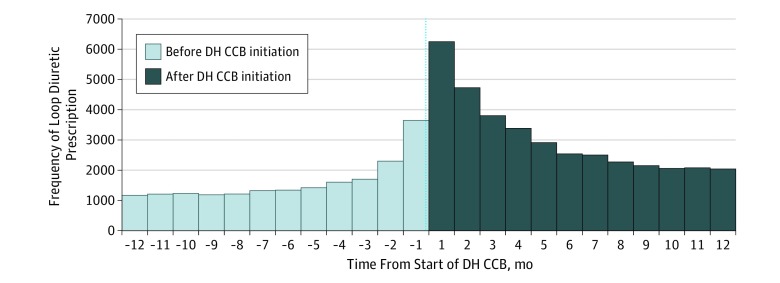
Prescription Sequence Symmetry of Initial Loop Diuretic Prescription Within 360 Days of Initial Dihydropyridine Calcium Channel Blocker (DH CCB) Prescription Among Patients Without Heart Failure

Among patients without heart failure who were initiated on both a DH CCB and a loop diuretic within the 720-day period, the loop diuretic was initiated nearly twice as often after DH CCB initiation than before (cSR, 1.91). Adjustment for secular prescribing trends attenuated the crude sequence ratio slightly (aSR, 1.87; 95% CI, 1.84-1.90) (Table 1). Excess initial loop diuretic use occurred primarily in the first 4 months after initial DH CCB initiation (Figure 2).

**Table 1.** Prescribing Order of Initial Loop Diuretic and Initial DH CCB

Characteristic	Patients, No.				Null-Effect Ratio	Crude Sequence Ratio	Adjusted Sequence Ratio (95% CI)
	All DH CCB Initiators	DH CCB Initiators Included in Analysis	Receiving Loop Diuretic				
			After DH CCB	Before DH CCB			
No heart failure	1 206 093	55 818	36 608	19 210	1.02	1.91	1.87 (1.84-1.90)
Age, y							
<65	893 433	33 100	21 662	11 438	1.02	1.89	1.85 (1.81-1.89)
≥65	312 660	22 718	14 946	7772	1.02	1.92	1.89 (1.84-1.94)
Sex							
Men	590 883	22 902	15 264	7638	1.02	2.00	1.96 (1.91-2.01)
Women	615 210	32 916	21 344	11 572	1.02	1.84	1.81 (1.77-1.85)
DH CCB type							
Amlodipine	1 088 931	49 930	32 875	17 055	1.02	1.93	1.89 (1.86-1.93)
Other^a^	117 162	5888	3733	2155	1.04	1.73	1.67 (1.59-1.76)
DH CCB dose^b^							
Low	175 641	6684	4189	2495	1.04	1.68	1.61 (1.53-1.69)
Standard	759 568	31 742	20 397	11 345	1.02	1.80	1.77 (1.73-1.81)
High	265 829	17 310	11 982	5328	1.02	2.25	2.20 (2.13-2.27)
Other antihypertensive medications, No.							
0-1	557 933	17 618	12 789	4829	1.04	2.65	2.55 (2.47-2.64)
2-3	561 782	29 587	19 105	10 482	1.02	1.82	1.79 (1.75-1.84)
≥4	86 378	8613	4714	3899	1.00	1.21	1.21 (1.16-1.26)

Abbreviation: DH CCB, dihydropyridine calcium channel blocker.

^a^ Includes nifedipine, felodipine, and isradipine.

^b^ Doses were missing for 5055 DH CCB initiators and 82 patients with a loop diuretic claim within 360 days of DH CCB claim.

In the stratified analyses, aSR was disproportionately higher among DH CCB initiators who used high doses (aSR, 2.20; 95% CI, 2.13-2.27), among those who were initially prescribed amlodipine (aSR, 1.89; 95% CI, 1.86-1.93), among men (aSR, 1.96; 95% CI, 1.91-2.01), and among those who used fewer (ie, 0-1) antihypertensive classes before the initiation of DH CCB (aSR, 2.55; 95% CI, 2.47-2.64). There was no discernable difference in aSRs between those younger than 65 years and those aged 65 years or older (aSR, 1.85 [95% CI, 1.81-1.89] vs 1.89 [95% CI, 1.84-1.94]) (Table 1).

Among DH CCB initiators, we estimated that 1.4% of patients experienced the prescribing cascade in the year after initiation. Among subpopulations, the estimated incidence of the prescribing cascade was highest among older adults (14 946 [2.3%] vs 21 662 [1.1%]), women (21 344 [1.6%] vs 15 264 [1.3%]), patients initially prescribed amlodipine compared with other DH CCBs (32 875 [1.5%] vs 3733 [1.4%]), patients initially prescribed a high-dose DH CCB compared with those initially prescribed a low-dose DH CCB (11 982 [2.5%] vs 4189 [1.0%]), and patients receiving 2 to 3 other antihypertensive medications compared with those receiving at least 4 (19 105 [1.5%] vs 4714 [0.9%]) (Table 2). There was no decrease in the prescribing cascade over the duration of the study period (2007-2010, 16 614 [1.5%]; 2011-2013, 11 041 [1.4%]; 2014-2016, 8953 [1.5%]) (Table 2).

**Table 2.** Estimated Percentage of Prescribing Cascade Among DH CCB Initiators, by Prespecified Subpopulations

Variable	DH CCB Initiators, No.	Patients With Loop Diuretics Initiated After DH CCB, No. /Patients With Loop Diuretics Initiated Before DH CCB, No.	Estimated Proportion of Patients With Prescribing Cascade, %^a^
Period^b^			
2007-2010	496 792	16 614/9190	1.49
2011-2013	389 015	11 041/5740	1.36
2014-2016	320 286	8953/4280	1.46
Age, y			
<65	893 433	21 662/11 438	1.14
≥65	312 660	14 946/7772	2.30
Sex			
Men	590 883	15 264/7638	1.29
Women	615 210	21 344/11 572	1.59
DH CCB type			
Amlodipine	1 088 931	32 875/17 055	1.45
Other^c^	117 162	3733/2155	1.35
DH CCB dose			
Low	175 641	4189/2495	0.96
Standard	759 568	20 397/11 345	1.19
High	265 829	11 982/5328	2.50
Other antihypertensive medications, No.			
0-1	557 933	12 789/4829	1.43
2-3	561 782	19 105/10 482	1.53
≥4	86 378	4714/3899	0.94

Abbreviation: DH CCB, dihydropyridine calcium channel blocker.

^a^ The difference between the sequence order (number of patients with an initial loop diuretic claim after DH CCB initiation minus the number of patients with an initial loop diuretic claim before DH CCB initiation) divided by the number of DH CCB new users.

^b^ Because we assessed 360 days before and after DH CCB initiation instead of 365 days before and after, we included 1688 patients from 2006 in 2007 and 1336 patients from 2017 in 2016.

^c^ Includes nifedipine, felodipine, and isradipine.

### Negative Control Analyses

**Figure 3.**
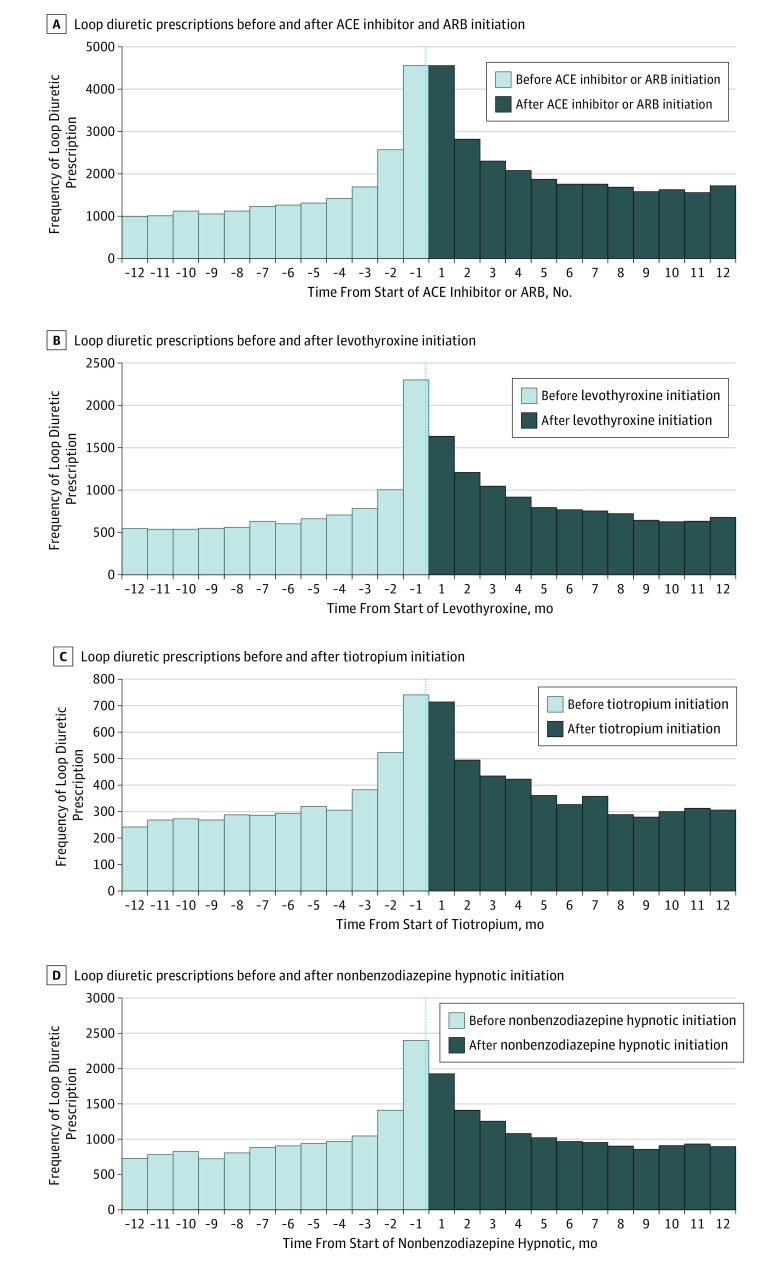
Prescription Sequence Symmetry of Initial Loop Diuretic Prescription Within 360 Days of Initial Negative Control Prescription Among Patients Without Congestive Heart Failure ACE indicates angiotensin-converting enzyme; ARB, angiotensin receptor blocker.

In the PSSAs, aSRs were significant for all negative controls but most prominent in ACE inhibitor or ARB negative controls and negligible in the other negative controls (Figure 3; eTable 3 in the Supplement). After adjusting for secular prescribing trends, initial loop diuretic use occurred more often after ACE inhibitor or ARB initiation than before (aSR, 1.27; 95% CI, 1.24-1.29) but to a lesser extent than after initiation of DH CCBs (aSR, 1.87; 95% CI, 1.84-1.90). On stratified analyses, there was a greater difference in aSRs for initial loop diuretic use following DH CCB compared with ACE inhibitor or ARB initiation among patients with 1 or fewer other antihypertensive medications (2.55 [95% CI, 2.47-2.64] vs 1.40 [95% CI, 1.36-1.43]) compared with the aSRs among patients with 2 or 3 other antihypertensive medications (1.79 [95% CI, 1.75-1.84] vs 0.99 [95% CI, 0.96-1.03]) and at least 4 other antihypertensive medications (1.21 [95% CI, 1.16-1.26] vs 0.80 [95% CI, 0.66-0.96]). This suggests that the interpretation of the prescribing cascade using a PSSA may be tempered by the general use of loop diuretics later in an antihypertensive regimen (ie, hypertension disease progression).

### Post Hoc Analysis

When restricting loop diuretic initiation to 180 days before and after DH CCB initiation, we found similar results (aSR, 1.88; 95% CI, 1.84-1.92) (eTable 4 in the Supplement). Using this period when analyzing ACE inhibitor or ARB as the negative control, the aSR was further attenuated (aSR, 1.12; 95% CI, 1.10-1.15). We also noted similar differences among subpopulations in the stratified analyses when restricting the period of loop diuretic initiation. The exception was age, in which patients aged 65 years or older had slightly higher aSRs compared with patients younger than 65 years (1.95 [95% CI, 1.88-2.01] vs 1.82 [95% CI, 1.77-1.87]) (eTable 4 in the Supplement).

## Discussion

Dihydropyridine calcium channel blockers are an important treatment option for patients with hypertension because of their demonstrated ability to reduce risk of stroke, cardiovascular events, and cardiovascular mortality^3,4,5^; however, continued use may be limited among some patients who develop edema, a well-known adverse event, which was described in a systematic review and meta-analysis of 92 randomized controlled trials.^10^ Unfortunately, the epidemiology of the prescribing cascade is not well documented.^14,53,54,55^ Beyond being described as a potential prescribing cascade in various reviews, published evidence is currently limited to 1 case report,^28^ which suggested the prescribing cascade may have contributed to a fall-related fracture and subsequent need for hospitalization and rehabilitation. Another study^29^ used cross-sectional data to estimate that the prescribing cascade impacted approximately 2.2 million patient visits in the United States per year using cross-sectional data.

Our findings support the existence of the prescribing cascade with an aSR of 1.87 (95% CI, 1.84-1.90). Moreover, we identified a higher aSR among men, patients initially prescribed amlodipine, patients initially prescribed a high-dose DH CCB, and patients receiving 1 or fewer other antihypertensive classes, suggesting a higher likelihood of inappropriately prescribed loop diuretics for edema within these subpopulations. Interestingly, we found similar aSRs when stratified between patients younger than 65 years (aSR, 1.85; 95% CI, 1.81-1.89) and 65 years or older (aSR, 1.89; 95% CI, 1.84-1.94); however, when the exposure window was restricted to 180 days before and after DH CCB initiation, the aSR was slightly higher in patients aged 65 or older (aSR, 1.95; 95% CI, 1.88-2.01) compared with those younger than 65 (aSR, 1.82; 95% CI, 1.71-1.87). This potentially suggests that inappropriate prescribing of loop diuretics for edema is similarly poor in younger adults when compared with older adults.

Overall, we identified an estimated incidence rate of the prescribing cascade to be 1.4% among DH CCB initiators; however, the incidence may be tempered given the significant results of the PSSA using negative controls (primarily ACE inhibitors or ARBs). Additionally, we estimated that 11.7% of patients who developed edema were treated with a loop diuretic, by dividing the adjusted prescribing cascade incidence in the present study (1.4%) by the estimated number of patients who developed DH CCB–induced edema as reported in a systematic review and meta-analysis (12.3%).^10,49^ This may suggest that approximately 1 in 9 patients who developed DH CCB–induced edema may have experienced a prescribing cascade.^56^ The estimated incidence of the prescribing cascade was doubled in patients aged 65 years or older compared with patients younger than 65 years (2.3% vs 1.1%). Given the similar aSRs between older and younger adults in the analysis of loop diuretic initiation 360 days before and after DH CCB initiation, the increased incidence is likely attributable to an increased presentation of edema among older adults.

We also identified no decreasing trend in the incidence of the prescribing cascade, suggesting a lack of improvement in recognizing the DH CCB adverse event over the 10-year study period. This differs from findings of a Dutch study,^49^ which identified a reduction in the incidence of an ACE inhibitor–induced cough-antitussive prescribing cascade during the study period. Thus, while awareness of ACE inhibitor–induced cough might have increased over time, leading to improvements in avoiding prescribing cascades, this has not been the case with the DH CCB prescribing cascade in the United States. Future studies are needed to test strategies to mitigate or prevent prescribing cascades.

It appears that patients are at highest risk of the prescribing cascade in the 4 months after initiation of DH CCBs; therefore, clinicians must pay particular attention to signs and symptoms of edema immediately after initiation. Dose reductions, especially when a high-dose DH CCB was used, or discontinuation should be recommended if edema develops, and an alternative antihypertensive should be used when necessary. Within our population, 31% of patients were initially prescribed high-dose DH CCBs and 85% of patients had used 3 or fewer antihypertensive medications before initiation of DH CCBs, suggesting dose reduction and use of alternative antihypertensive medications were available for most patients.

A benefit of using a self-controlled analysis like the PSSA is that it avoids the need to adjust for time-invariant confounders; however, it is still subject to biases, such as secular trends in prescribing (ie, secular changes in the prescribing of DH CCBs and loop diuretics over time) and within-patient, time-varying biases (ie, increased use with hypertension disease progression).^48^ We addressed secular trends in prescribing by calculating aSRs using the null-effect ratio and found limited effects. Additionally, we used a 720-day period to limit within-patient, time-varying biases; in post hoc analyses, we further restricted exposures to a 360-day period, which resulted in a further reduction in aSRs among ACE inhibitors or ARBs with similar aSRs for DH CCBs.

Innovative to our analysis was the incorporation of negative controls and stratified analyses by the number of unique antihypertensive classes before initiation of DH CCBs. The evaluation of aSRs among ACE inhibitor and ARB initiators helped quantify the influence of natural disease progression that may warrant the use of a loop diuretic for hypertension control. Likewise, stratified analysis allowed the examination of patients who had exhausted a larger number of first-line antihypertensive treatments, which may warrant the use of a loop diuretic for antihypertensive treatment. Similar to our analysis of DH CCB initiators, we found excess initiation of loop diuretics following ACE inhibitor or ARB initiation but to a much lesser extent. Thus, under the assumption that DH CCB initiators and ACE inhibitor or ARB initiators share similar trajectories in hypertension progression that would warrant the use of loop diuretics, our use of negative controls cannot fully explain the aSR among DH CCB users and further suggests the presence of a prescribing cascade in this population.

Also, as hypothesized, we identified disproportionately higher prescriptions of loop diuretics after the initiation of high-dose DH CCBs and within only a few months after the initiation of DH CCBs. Both the noted dose-response association and the proximity between DH CCB and loop diuretic initiation suggests the potential presence of a causal effect (ie, development of edema secondary to DH CCB exposure and subsequent treatment with loop diuretic).

### Limitations

There are several limitations to note. Certain loop diuretics may be available at community pharmacies at prices below insurance copayments. This may result in missing claims if loop diuretics were paid for out of pocket. Patients who developed DH CCB–induced edema may have been misdiagnosed as having heart failure within 360 days before or after initiation of DH CCBs and were therefore excluded; on the other hand, patients with heart failure may not have been diagnosed (although the diagnosis of heart failure has been validated with specificity of ≥95%),^57^ also resulting in the potential for misclassification. The diagnosis of edema has not been validated and is likely underdocumented; therefore, we incorporated negative controls, restricted exposure windows, and stratified on number of antihypertensive medications to evaluate excess loop diuretic use in the context of other valid indications.

## Conclusions

In summary, despite edema being a well-known adverse effect of DH CCBs, our findings suggest that an appreciable proportion of patients received loop diuretics instead of DH CCB dose reduction or discontinuation. This occurred more commonly among patients who were initially prescribed high-dose DH CCBs. Individuals initiating DH CCBs were at highest risk for the prescribing cascade in the first 4 months following initiation, suggesting the need to evaluate for edema during early follow-up visits. Given that loop diuretics are among the medications most frequently associated with adverse events, especially in older adults, subsequent research is needed to measure downstream consequences of this prescribing cascade, such as fall-related injuries, increased health care utilization, and increased costs.

## Notes

Supplement. **eTable 1. **Diagnosis Codes for Heart Failure **eTable 2. **Dose Comparison of Calcium Channel Blockers **eTable 3. **Baseline Characteristics of Patients Without Heart Failure Initiated on Dihydropyridine Calcium Channel Blocker Who Were Included vs Excluded in Prescription Sequence Symmetry Analysis **eTable 4. **Prescribing Order of Initial Loop Diuretic Restricted to Within 180 Days of Initial Dihydropyridine Calcium Channel Blocker **eTable 5. **Prescribing Order of Initial Loop Diuretic Prescription in Relation to Initial Prescriptions of Negative Controls

## References

[1] Benjamin EJ, Muntner P, Alonso A, ; American Heart Association Council on Epidemiology and Prevention Statistics Committee and Stroke Statistics Subcommittee Heart disease and stroke statistics 2019 update: a report from the American Heart Association. Circulation. 2019;139(10):-. doi:10.1161/CIR.0000000000000659 30700139

[2] Gu Q, Burt VL, Dillon CF, Yoon S Trends in antihypertensive medication use and blood pressure control among United States adults with hypertension: the National Health And Nutrition Examination Survey, 2001 to 2010. Circulation. 2012;126(17):2105-2114. doi:10.1161/CIRCULATIONAHA.112.096156 23091084

[3] Neal B, MacMahon S, Chapman N; Blood Pressure Lowering Treatment Trialists’ Collaboration Effects of ACE inhibitors, calcium antagonists, and other blood-pressure-lowering drugs: results of prospectively designed overviews of randomised trials: Blood Pressure Lowering Treatment Trialists’ Collaboration. Lancet. 2000;356(9246):1955-1964. doi:10.1016/S0140-6736(00)03307-9 11130523

[4] Pitt B, Byington RP, Furberg CD, ; PREVENT Investigators Effect of amlodipine on the progression of atherosclerosis and the occurrence of clinical events. Circulation. 2000;102(13):1503-1510. doi:10.1161/01.CIR.102.13.1503 11004140

[5] Staessen JA, Fagard R, Thijs L, ; The Systolic Hypertension in Europe (Syst-Eur) Trial Investigators Randomised double-blind comparison of placebo and active treatment for older patients with isolated systolic hypertension. Lancet. 1997;350(9080):757-764. doi:10.1016/S0140-6736(97)05381-6 9297994

[6] Whelton PK, Carey RM, Aronow WS, 2017 ACC/AHA/AAPA/ABC/ACPM/AGS/APhA/ASH/ASPC/NMA/PCNA guideline for the prevention, detection, evaluation, and management of high blood pressure in adults: a report of the American College of Cardiology/American Heart Association Task Force on Clinical Practice Guidelines. Hypertension. 2018;71(6):e13-e115. 29133356 10.1161/HYP.0000000000000065

[7] Mann JFE, Hilgers KF Use of thiazide diuretics in patients with primary (essential) hypertension. 2017 https://www.uptodate.com/contents/use-of-thiazide-diuretics-in-patients-with-primary-essential-hypertension. Accessed October 31, 2019.

[8] Li EC, Heran BS, Wright JM Angiotensin converting enzyme (ACE) inhibitors versus angiotensin receptor blockers for primary hypertension. Cochrane Database Syst Rev. 2014;(8):CD009096. doi:10.1002/14651858.CD009096.pub2 25148386 PMC6486121

[9] Caballero-Gonzalez FJ Calcium channel blockers in the management of hypertension in the elderly. Cardiovasc Hematol Agents Med Chem. 2015;12(3):160-165. doi:10.2174/1871525713666150310111554 25761102

[10] Makani H, Bangalore S, Romero J, Peripheral edema associated with calcium channel blockers: incidence and withdrawal rate: a meta-analysis of randomized trials. J Hypertens. 2011;29(7):1270-1280. doi:10.1097/HJH.0b013e3283472643 21558959

[11] Messerli FH Vasodilatory edema: a common side effect of antihypertensive therapy. Am J Hypertens. 2001;14(9, pt 1):978-979. doi:10.1016/S0895-7061(01)02178-1 11587169

[12] Messerli FH Vasodilatory edema: a common side effect of antihypertensive therapy. Curr Cardiol Rep. 2002;4(6):479-482. doi:10.1007/s11886-002-0110-9 12379167

[13] Sica D Calcium channel blocker-related peripheral edema: can it be resolved? J Clin Hypertens (Greenwich). 2003;5(4):291-294, 297. doi:10.1111/j.1524-6175.2003.02402.x 12939574 PMC8099365

[14] Krishnaswami A, Steinman MA, Goyal P, ; Geriatric Cardiology Section Leadership Council, American College of Cardiology Deprescribing in older adults with cardiovascular disease. J Am Coll Cardiol. 2019;73(20):2584-2595. doi:10.1016/j.jacc.2019.03.467 31118153 PMC6724706

[15] Rochon PA, Gurwitz JH Optimising drug treatment for elderly people: the prescribing cascade. BMJ. 1997;315(7115):1096-1099. doi:10.1136/bmj.315.7115.1096 9366745 PMC2127690

[16] McCarthy LM, Visentin JD, Rochon PA Assessing the scope and appropriateness of prescribing cascades. J Am Geriatr Soc. 2019;67(5):1023-1026. doi:10.1111/jgs.15800 30747997

[17] Claxton AJ, Cramer J, Pierce C A systematic review of the associations between dose regimens and medication compliance. Clin Ther. 2001;23(8):1296-1310. doi:10.1016/S0149-2918(01)80109-0 11558866

[18] Patel M, Vellanki K, Leehey DJ, Urinary incontinence and diuretic avoidance among adults with chronic kidney disease. Int Urol Nephrol. 2016;48(8):1321-1326. doi:10.1007/s11255-016-1304-1 27209426

[19] van Kraaij DJ, Haagsma CJ, Go IH, Gribnau FW Drug use and adverse drug reactions in 105 elderly patients admitted to a general medical ward. Neth J Med. 1994;44(5):166-173. doi:10.1016/0300-2977(95)90003-9 8028691

[20] Cheung CM, Ponnusamy A, Anderton JG Management of acute renal failure in the elderly patient: a clinician’s guide. Drugs Aging. 2008;25(6):455-476. doi:10.2165/00002512-200825060-00002 18540687

[21] Tannenbaum C, Johnell K Managing therapeutic competition in patients with heart failure, lower urinary tract symptoms and incontinence. Drugs Aging. 2014;31(2):93-101. doi:10.1007/s40266-013-0145-1 24357134 PMC3907694

[22] Sica DA Diuretic-related side effects: development and treatment. J Clin Hypertens (Greenwich). 2004;6(9):532-540. doi:10.1111/j.1524-6175.2004.03789.x 15365284 PMC8109680

[23] Berry SD, Mittleman MA, Zhang Y, New loop diuretic prescriptions may be an acute risk factor for falls in the nursing home. Pharmacoepidemiol Drug Saf. 2012;21(5):560-563. doi:10.1002/pds.3256 22422651 PMC3330142

[24] Kelly J, Chamber J Inappropriate use of loop diuretics in elderly patients. Age Ageing. 2000;29(6):489-493. doi:10.1093/ageing/29.6.489 11191239

[25] Wehling M Morbus diureticus in the elderly: epidemic overuse of a widely applied group of drugs. J Am Med Dir Assoc. 2013;14(6):437-442. doi:10.1016/j.jamda.2013.02.002 23510827

[26] Corrao G, Mazzola P, Monzio Compagnoni M, Antihypertensive medications, loop diuretics, and risk of hip fracture in the elderly: a population-based cohort study of 81,617 Italian patients newly treated between 2005 and 2009. Drugs Aging. 2015;32(11):927-936. doi:10.1007/s40266-015-0306-5 26589307

[27] Berry SD, Zhu Y, Choi H, Kiel DP, Zhang Y Diuretic initiation and the acute risk of hip fracture. Osteoporos Int. 2013;24(2):689-695. doi:10.1007/s00198-012-2053-3 22790610 PMC3594771

[28] Nguyen PV, Spinelli C Prescribing cascade in an elderly woman. Can Pharm J (Ott). 2016;149(3):122-124. doi:10.1177/1715163516640811 27212961 PMC4860747

[29] Vouri SM, van Tuyl JS, Olsen MA, Xian H, Schootman M An evaluation of a potential calcium channel blocker-lower-extremity edema-loop diuretic prescribing cascade. J Am Pharm Assoc (2003). 2018;58(5):534-539.e4. doi:10.1016/j.japh.2018.06.014 30033126 PMC6424490

[30] DeRhodes KH The dangers of ignoring the Beers criteria: the prescribing cascade. JAMA Intern Med. 2019;179(7):863-864. doi:10.1001/jamainternmed.2019.1288 31081879

[31] Hallas J Evidence of depression provoked by cardiovascular medication: a prescription sequence symmetry analysis. Epidemiology. 1996;7(5):478-484. doi:10.1097/00001648-199609000-00005 8862977

[32] Wahab IA, Pratt NL, Wiese MD, Kalisch LM, Roughead EE The validity of sequence symmetry analysis (SSA) for adverse drug reaction signal detection. Pharmacoepidemiol Drug Saf. 2013;22(5):496-502. doi:10.1002/pds.3417 23412832

[33] Lai EC, Pratt N, Hsieh CY, Sequence symmetry analysis in pharmacovigilance and pharmacoepidemiologic studies. Eur J Epidemiol. 2017;32(7):567-582. doi:10.1007/s10654-017-0281-8 28698923

[34] Lai EC, Yang YH, Lin SJ, Hsieh CY Use of antiepileptic drugs and risk of hypothyroidism. Pharmacoepidemiol Drug Saf. 2013;22(10):1071-1079. doi:10.1002/pds.3498 23946049

[35] Hallas J, Pottegård A Use of self-controlled designs in pharmacoepidemiology. J Intern Med. 2014;275(6):581-589. doi:10.1111/joim.12186 24635348

[36] von Elm E, Altman DG, Egger M, Pocock SJ, Gøtzsche PC, Vandenbroucke JP; STROBE Initiative The Strengthening the Reporting of Observational Studies in Epidemiology (STROBE) statement: guidelines for reporting observational studies. Lancet. 2007;370(9596):1453-1457. doi:10.1016/S0140-6736(07)61602-X 18064739

[37] Ray WA Evaluating medication effects outside of clinical trials: new-user designs. Am J Epidemiol. 2003;158(9):915-920. doi:10.1093/aje/kwg231 14585769

[38] Healthcare Cost and Utilization Project Elixhauser comorbidity software version 3.7. https://www.hcup-us.ahrq.gov/toolssoftware/comorbidity/comorbidity.jsp. Accessed November 18, 2019.

[39] Healthcare Cost and Utilization Project Elixhauser Comorbidity Software for *ICD-10-CM* https://www.hcup-us.ahrq.gov/toolssoftware/comorbidityicd10/comorbidity_icd10.jsp. Accessed November 18, 2019.

[40] Pratt N, Chan EW, Choi NK, Prescription sequence symmetry analysis: assessing risk, temporality, and consistency for adverse drug reactions across datasets in five countries. Pharmacoepidemiol Drug Saf. 2015;24(8):858-864. doi:10.1002/pds.3780 25907076 PMC4690514

[41] Vegter S, de Jong-van den Berg LT Misdiagnosis and mistreatment of a common side-effect: angiotensin-converting enzyme inhibitor-induced cough. Br J Clin Pharmacol. 2010;69(2):200-203. doi:10.1111/j.1365-2125.2009.03571.x 20233184 PMC2824482

[42] van Boven JF, de Jong-van den Berg LT, Vegter S Inhaled corticosteroids and the occurrence of oral candidiasis: a prescription sequence symmetry analysis. Drug Saf. 2013;36(4):231-236. doi:10.1007/s40264-013-0029-7 23516006

[43] de la Sierra A Mitigation of calcium channel blocker-related oedema in hypertension by antagonists of the renin-angiotensin system. J Hum Hypertens. 2009;23(8):503-511. doi:10.1038/jhh.2008.157 19148104

[44] He T, Liu X, Li Y, High-dose calcium channel blocker (CCB) monotherapy vs combination therapy of standard-dose CCBs and angiotensin receptor blockers for hypertension: a meta-analysis. J Hum Hypertens. 2017;31(2):79-88. doi:10.1038/jhh.2016.46 27511478

[45] Park JB, Ha JW, Jung HO, Rhee MY; FOCUS Investigators Randomized trial comparing the effects of a low-dose combination of nifedipine GITS and valsartan versus high-dose monotherapy on central hemodynamics in patients with inadequately controlled hypertension: FOCUS Study. Blood Press Monit. 2014;19(5):294-301. doi:10.1097/MBP.0000000000000061 24915052

[46] Hernández RH, Armas-Hernández MJ, Chourio JA, Comparative effects of amlodipine and nifedipine GITS during treatment and after missing two doses. Blood Press Monit. 2001;6(1):47-57. doi:10.1097/00126097-200102000-00008 11248761

[47] Lefebvre J, Poirier L, Archambault F, Jewell D, Reed CV, Lacourcière Y Comparative effects of felodipine ER, amlodipine and nifedipine GITS on 24 h blood pressure control and trough to peak ratios in mild to moderate ambulatory hypertension: a forced titration study. Can J Cardiol. 1998;14(5):682-688. 9627524

[48] Singh H, Johnson ML Prescribing patterns of diuretics in multi-drug antihypertensive regimens. J Clin Hypertens (Greenwich). 2005;7(2):81-87. doi:10.1111/j.1524-6175.2005.03922.x 15722652 PMC8109453

[49] Vegter S, de Boer P, van Dijk KW, Visser S, de Jong-van den Berg LT The effects of antitussive treatment of ACE inhibitor-induced cough on therapy compliance: a prescription sequence symmetry analysis. Drug Saf. 2013;36(6):435-439. doi:10.1007/s40264-013-0024-z 23494997

[50] Takeuchi Y, Shinozaki T, Matsuyama Y A comparison of estimators from self-controlled case series, case-crossover design, and sequence symmetry analysis for pharmacoepidemiological studies. BMC Med Res Methodol. 2018;18(1):4. doi:10.1186/s12874-017-0457-7 29310575 PMC5759844

[51] Kalisch Ellett LM, Pratt NL, Barratt JD, Rowett D, Roughead EE Risk of medication-associated initiation of oxybutynin in elderly men and women. J Am Geriatr Soc. 2014;62(4):690-695. doi:10.1111/jgs.12741 24635879

[52] Garrison SR, Dormuth CR, Morrow RL, Carney GA, Khan KM Nocturnal leg cramps and prescription use that precedes them: a sequence symmetry analysis. Arch Intern Med. 2012;172(2):120-126. doi:10.1001/archinternmed.2011.1029 22157068

[53] Pollock M, Bazaldua OV, Dobbie AE Appropriate prescribing of medications: an eight-step approach. Am Fam Physician. 2007;75(2):231-236. 17263218

[54] Bazaldua O, Pollack M, Roaten S, Dobbie A Teaching the ESSEnCE of office-based prescribing. Fam Med. 2006;38(5):316-318. 16673188

[55] Balducci L, Goetz-Parten D, Steinman MA Polypharmacy and the management of the older cancer patient. Ann Oncol. 2013;24(suppl 7):vii36-vii40. doi:10.1093/annonc/mdt266 24001761 PMC6278993

[56] Hellfritzsch M, Rasmussen L, Hallas J, Pottegård A Using the symmetry analysis design to screen for adverse effects of non-vitamin K antagonist oral anticoagulants. Drug Saf. 2018;41(7):685-695. doi:10.1007/s40264-018-0650-6 29498009

[57] McCormick N, Lacaille D, Bhole V, Avina-Zubieta JA Validity of heart failure diagnoses in administrative databases: a systematic review and meta-analysis. PLoS One. 2014;9(8):e104519. doi:10.1371/journal.pone.0104519 25126761 PMC4134216

