# Supplementary material for: Magnitude of and Characteristics Associated With the Treatment of Calcium Channel Blocker–Induced Lower-Extremity Edema With Loop Diuretics
Source: JAMA Netw Open. 2019 Dec 27;2(12):e1918425. doi: 10.1001/jamanetworkopen.2019.18425 (PMC6991233; doi:10.1001/jamanetworkopen.2019.18425)
Supplement: Supplement. — eTable 1. Diagnosis Codes for Heart Failure eTable 2. Dose Comparison of Calcium Channel Blockers eTable 3. Baseline Characteristics of Patients Without Heart Failure Initiated on Dihydropyridine Calcium Channel Blocker Who Were Included vs Excluded in Prescription Sequence Symmetry Analysis eTable 4. Prescribing Order of Initial Loop Diuretic Restricted to Within 180 Days of Initial Dihydropyridine Calcium Channel Blocker eTable 5. Prescribing Order of Initial Loop Diuretic Prescription in Relation to Initial Prescriptions of Negative Controls [file jamanetwopen-2-e1918425-s001.pdf]

Supplementary Online Content

Vouri SM, Jiang X, Manini TM, et al. Magnitude of and characteristics associated with the treatment of calcium channel blocker–induced lower-extremity edema with loop diuretics. *JAMA Netw Open*. 2019;2(12):e1918425. doi:10.1001/jamanetworkopen.2019.18425

eTable 1. Diagnosis Codes for Heart Failure

eTable 2. Dose Comparison of Calcium Channel Blockers

eTable 3. Baseline Characteristics of Patients Without Heart Failure Initiated on Dihydropyridine Calcium Channel Blocker Who Were Included vs Excluded in Prescription Sequence Symmetry Analysis

eTable 4. Prescribing Order of Initial Loop Diuretic Restricted to Within 180 Days of Initial Dihydropyridine Calcium Channel Blocker

eTable 5. Prescribing Order of Initial Loop Diuretic Prescription in Relation to Initial Prescriptions of Negative Controls

This supplementary material has been provided by the authors to give readers additional information about their work.

© 2019 Vouri SM et al. *JAMA Network Open.*

eTable 1. Diagnosis Codes for Heart Failure

Diagnosis Codes	Heart Failure
**ICD-9-CM Codes**	"39891", "428", "40201", "40211", "40291", "40401", "40411", "40491", "40403", "40413", "40493"
**ICD-10 Codes**	"I0981", "I501", "I5020", "I5021", "I5022", "I5023", "I5030", "I5031", "I5032", "I5033", "I5040", "I5041", "I5042", "I5043", "I50810", "I50811", "I50812", "I50813", "I50814", "I5082", "I5083", "I5084", "I5089", "I509", "I110", "I130", "I132"
Abbreviation: CM, Clinical Modification; ICD, International Classification of Disease;	

© 2019 Vouri SM et al. *JAMA Network Open.*

eTable 2. Dose Comparison of Calcium Channel Blockers

Dose	Dihydropyridine Calcium Channel Blockers			
	**Amlodipine**	**Nifedipine**	**Felodipine**	**Isradipine**
**Low Dose**	<5 mg/day	<30mg/day	<5 mg/day	<5 mg/day
**Standard Dose**	≥ 5mg/day & <10mg/day	≥ 30mg/day & <60mg/day	≥ 5mg/day & <10mg/day	≥ 5mg/day & <10mg/day
**High Dose**	≥ 10mg/day	≥ 60mg/day	≥ 10mg/day	≥ 10mg/day

© 2019 Vouri SM et al. *JAMA Network Open.*

eTable 3. Baseline Characteristics of Patients Without Heart Failure Initiated on Dihydropyridine Calcium Channel Blocker Who Were Included vs Excluded in Prescription Sequence Symmetry Analysis

Variables	DH-CCB Initiators	
	Included	Excluded
	**Patients Initiated on Loop Diuretic ± 360 days of DH-CCB (n=55,818)**	**Patients Not Initiated on Loop Diuretic ± 360 days of DH-CCB (n=1,150,275)**
	n (%)	
**Years**		
2007-2010	25,804 (46.2)	470,988 (40,9)
2011-2013	16,781 (30.1)	372,234 (32.4)
2014-2016	13,233 (23.7)	307,053 (26.7)
**Age**		
<65 years	33,100 (59.3)	860,333 (74.8)
≥65 years	22,718 (40.7)	289,942 (25.2)
**Sex**		
Men	22,902 (41.0)	567,981 (49.4)
Women	32,916 (59.0)	582,294 (50.6)
**DH-CCB Type**		
Amlodipine	49,930 (89.5)	1,039,001 (90.3)
Other DH-CCB[b]	5,888 (10.5)	111,274 (0,7)
**DH-CCB Dose**		
Low Dose	6,684 (12.0)	168,957 (14.7)
Standard Dose	31,742 (56.9)	727,826 (63.3)
High Dose	17,310 (31.0)	248,519 (21.6)
Missing Dose	82 (0.1)	4,973 (0.4)
**Number of Other Antihypertensives**		
0-1	17,618 (31.6)	540,315 (47.0)
2-3	29,587 (53.0)	532,195 (46.3)
≥4	8,613 (15.4)	77,765 (6.8)

[a] Since we assessed 360 days before and after DH-CCB instead of 365 days before and after DH-CCB, we included patients from 2006 into 2007 and 1,336 from 2017 into 2016.
[b] Other DH-CCB includes nifedipine, felodipine, isradipine
Abbreviation: CI, confidence interval; DH-CCB, dihydropyridine calcium channel blocker

© 2019 Vouri SM et al. *JAMA Network Open.*

eTable 4. Prescribing Order of Initial Loop Diuretic Restricted to Within 180 Days of Initial Dihydropyridine Calcium Channel Blocker

DH-CCB	DH-CCB Initiators (n)	Total number (n)	Number of Patients on Loop Diuretic (n)		Null-effect ratio	Crude Sequence Ratio	Adjusted Sequence Ratio (95% CI)
			After DH-CCB	Before DH-CCB			
**Excluding Heart Failure**	1,206,093	35,506	23,550	11,956	1.05	1.97	1.88 (1.84-1.92)
**Age**							
<65 years	893,433	21,266	13,997	7,269	1.06	1.93	1.82 (1.77-1.87)
≥65 years	312,660	14,240	9,553	4,687	1.05	2.04	1.95 (1.88-2.01)
**Sex**							
Men	590,883	14,637	9,730	4,907	1.04	1.98	1.91 (1.85-1.98)
Women	615,210	20,869	13,820	7,049	1.06	1.96	1.85 (1.80-1.90)
**DH-CCB Type**							
Amlodipine	1,088,931	31,547	20,989	10,558	1.05	1.99	1.90 (1.85-1.94)
Other	117,162	3,959	2,561	1,398	1.07	1.83	1.71 (1.60-1.83)
**DH-CCB Dose**[a]							
Low Dose	175,641	3,988	2,514	1,474	1.06	1.71	1.60 (1.50-1.71)
Standard Dose	759,568	19,506	12,594	6,912	1.04	1.82	1.76 (1.71-1.81)
High Dose	265,829	11,960	8,415	3,545	1.08	2.37	2.21 (2.12-2.29)
**Number of Other Antihypertensives**							
0-1	557,933	11,642	8,381	3,261	1.12	2.57	2.30 (2.21-2.40)
2-3	561,782	18,457	12,083	6,374	1.04	1.90	1.83 (1.77-1.88)
≥4	86,378	5,407	3,086	2,321	1.02	1.33	1.31 (1.24-1.38)

[a] Doses were missing for DH-CCB initiators (n=5,055) and patients with loop diuretic claim within 180 days of DH-CCB claim (n=52)
[b] Other DH-CCB includes nifedipine, felodipine, isradipine
Abbreviation: CI, confidence interval; DH-CCB, dihydropyridine calcium channel blocker

© 2019 Vouri SM et al. *JAMA Network Open.*

eTable 5. Prescribing Order of Initial Loop Diuretic Prescription in Relation to Initial Prescriptions of Negative Controls

	Initiators (n)	Total number (n)	Number of Patients on Loop Diuretic (n)		Null-effect ratio	Crude Sequence Ratio	Adjusted Sequence Ratio (95% CI)
			After Negative Control	Before Negative Control			
ACE Inhibitor or ARB							
**Overall**	1,801,766	44,627	25,273	19,354	1.03	1.31	1.27 (1.24-1.29)
**Age**							
<65 years	1,540,132	30,980	17,622	13,358	1.04	1.46	1.27 (1.24-1.30)
≥65 years	261,634	13,647	7,651	5,996	1.02	1.40	1.25 (1.21-1.29)
**Number of Other Antihypertensives**							
0-1	1,545,202	32,467	19,206	13,261	1.04	1.45	1.40 (1.36-1.43)
2-3	252,174	11,718	5,873	5,873	1.01	1.00	0.99 (0.96-1.03)
≥4	4,390	442	194	248	0.98	0.78	0.80 (0.66-0.96)
Levothyroxine							
**Overall**	729,010	19,759	10,387	9,372	1.02	1.11	1.09 (1.06-1.12)
**Age**							
<65 years	613,318	13,175	7,047	6,128	1.03	1.15	1.12 (1.08-1.16)
≥65 years	115,692	6,584	3,340	3,244	1.01	1.03	1.02 (0.97-1.07)
**Number of Other Antihypertensives**							
0-1	583,692	11,403	6,587	4,816	1.03	1.37	1.32 (1.27-1.37)
2-3	132,,948	7,126	3,326	3,800	1.00	0.87	0.87 (0.83-0.91)
≥4	12370	1,230	474	756	0.98	0.63	0.64 (0.57-0.72)
Tiotropium							
**Overall**	134,903	8,768	4,583	4,185	1.01	1.10	1.08 (1.04-1.13)
**Age**							
<65 years	78,388	4,026	2,034	1,992	1.01	1.02	1.01 (0.95-1.07)
≥65 years	56,545	4,742	2,549	2,193	1.01	1.16	1.15 (1.08-1.22)
**Number of Other Antihypertensives**							

© 2019 Vouri SM et al. *JAMA Network Open.*

0-1	85,725	4,064	2,305	1,759	1.02	1.31	1.29 (1.21-1.37)
2-3	43,440	3,892	1,940	1,952	1.01	0.99	0.99 (0.93-1.05)
≥4	5,738	812	338	474	1.00	0.71	0.71 (0.62-0.82)
Non-Benzodiazepine Hypnotics							
**Overall**	1,018,185	25,519	13,094	124,25	1.01	1.05	1.04 (1.02-1.07)
**Age**							
<65 years	897,262	17,859	9,285	8,574	1.01	1.08	1.07 (1.04-1.10)
≥65 years	120,923	7,660	3,809	3,851	1.00	0.99	0.99 (0.94-1.03)
**Number of Other Antihypertensives**							
0-1	812,836	13,506	7,723	5,783	1.02	1.34	1.31 (1.26-1.35)
2-3	186,015	10,020	4,565	5,455	1.00	0.84	0.84 (0.81-0.87)
≥4	19,334	1,993	806	1,187	0.98	0.68	0.69 (0.63-0.75)

Abbreviations: ACE, angiotensin converting enzyme; ARB, angiotensin receptor blocker

[a] Excludes patients diagnosed with heart failure or filled a dihydropyridine calcium channel blocker claim ±360 days of the negative control index drug

© 2019 Vouri SM et al. *JAMA Network Open.*
